# Concomitant detection of hematological neoplasm and carcinoma: report on seven cases

**DOI:** 10.1590/S1516-31802008000400013

**Published:** 2008-07-03

**Authors:** Mariana Montenegro de Melo Lira, Luciana Regina Moreira, Thais Ruano, Maísa Momesso Quintal, Rita de Cássia Perina Martins, Annacarolina Fabiana Lúcia da Silva, Fernando Augusto Soares, José Vassallo

**Keywords:** Neoplasms, multiple primary, Lymphoma, non-Hodgkin's, Carcinoma, Immunohistochemistry, Pathology, Neoplasias sincrônicas, Linfoma não Hodgkin, Carcinoma, Imunohistoquímica, Patologia

## Abstract

**CONTEXT:**

The presence of multiple neoplasms in one patient is an uncommon event. Its occurrence ranges from 1.2% to 4.5% of cancer patients in autopsy and clinical studies. In the present article, cases of synchronous diagnoses of carcinoma and lymphoid neoplasms are reported. The intention of this report was to alert clinicians and pathologists to the possibility of the existence of concomitant neoplasms, in order to prevent inaccurate or delayed diagnosis and staging.

**CASES:**

Seven patients (four female and three male) with a median age of 61.4 years were diagnosed as having concomitant epithelial and hematological neoplasms.

**DISCUSSION AND CONCLUSION:**

Lymph nodes should be carefully examined when searching for metastases, because of the possibility of a second hematological malignancy. Whenever uncommon suspicious morphological features are seen in such neoplasms, an immunohistochemical analysis is essential.

## RESUMO

**CONTEXTO:**

A presença de múltiplas neoplasias em um mesmo paciente é evento infreqüente, correspondendo a 1,2%-4,5% dos pacientes com câncer em estudos clínicos e de autópsias. Neste artigo, os autores relatam casos de diagnósticos sincrônicos de carcinomas e neoplasias linfóides. Ao apresentar estes casos, tenciona-se alertar clínicos e patologistas para a possibilidade da ocorrência de neoplasias concomitantes, para evitar atrasos ou erros diagnósticos ou de estadiamento da doença.

**SÉRIE DE CASOS:**

Sete pacientes, quatro mulheres e três homens, com média de idade de 61,4 anos, foram diagnosticados com neoplasias epitelial e hematológica concomitantes.

**DISCUSSÃO E CONCLUSÃO:**

Os linfonodos devem ser cuidadosamente examinados quando se buscam metástases, dada a possibilidade de uma neoplasia hematológica concomitante. Sempre que aspectos morfológicos suspeitos forem detectados, um estudo imunoistoquímico faz-se necessário.

## INTRODUCTION

The presence of multiple neoplasms in one patient is an uncommon event. Its occurrence ranges from 1.2% to 4.5% of cancer patients in autopsy and clinical studies.^1^ Different neoplasms may be synchronous, when separated by an interval shorter than six months, or metachronous, when separated by a greater interval.^1^ It is generally easier to detect metachronous multiple neoplasia than to detect the synchronous type, since metachronous neoplasia produces signs and symptoms that are distinct in time and anatomical site in patients who are under close supervision to guard against recurrence of their initial neoplasm. Occasionally, however, the presence of another malignancy is an unexpected finding during the follow-up for a previously diagnosed neoplasm. Such diagnoses represent challenges for the practitioner, and the presence of an unexpected second malignancy may pose dilemmas with regard to diagnosis and treatment.^1^

## OBJECTIVE

The rarity of occurrences of multiple neoplasms and the need to alert both pathologists and clinicians regarding this possibility in daily practice prompted us to report on seven such cases diagnosed in our service.

## METHODS

This was a descriptive retrospective study on cancer patients diagnosed at the university hospital of Universidade Estadual de Campinas (Unicamp) and at Hospital do Câncer A. C. Camargo, São Paulo, Brazil. The patient sample consisted of seven cases of concomitant detection of hematological neoplasm and carcinoma.

Biopsy and surgical specimens from these patients were fixed in 10% formalin and embedded in paraffin. Immunohistochemical analysis was performed using antibodies for epithelial and lymphoid markers, all provided by Dakocytomation, Carpenteria, California, United States. Heat-induced antigen retrieval was achieved using a steamer. The samples were developed using the EnVision peroxidase technique (Dakopatts, code K1491).

## RESULTS

**Figure 1:**
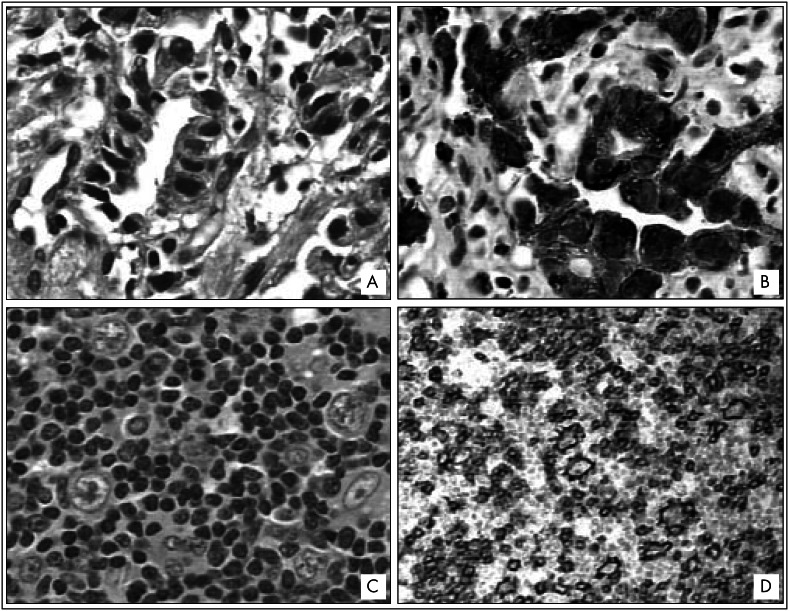
A = Gastric adenocarcinoma, intestinal type (hematoxylin-eosin, 400 x). B = Immunostaining for pan-cytokeratin AE1/AE3 showing positivity in neoplastic cells (immunoperoxidase, anti-cytokeratin, 400 x). C = Nodular lymphocyte-predominant Hodgkin's lymphoma in a perigastric lymph node (hematoxylin-eosin, 400 x). D = Immunostaining for CD 20 showing positivity in neoplastic cells and in nodules of small reactive lymphocytes (immunoperoxidase, anti-CD20, 100 x).

Data on all the patients were obtained from their clinical files and are summarized in Table 1. Four patients were female and three, male. Their ages ranged from 16 to 79 years (mean of 61.4 years). In cases 1, 2 and 3, the diagnosis of the two neoplasms was made simultaneously, while in cases 4 and 6, carcinoma was diagnosed first, and in cases 5 and 7, the hematological neoplasm was diagnosed first. All of the diagnoses were confirmed by histopathological and immunohistochemical analysis. One example of simultaneous infiltration of lymph nodes by Hodgkin's lymphoma and metastatic carcinoma cells (case 1) is shown in Figure 1.

**Table 1:** Data from seven patients with synchronous lymphoma and carcinoma

Case	Age (years)	Gender	Hematological neoplasm	Carcinoma	Therapy	Follow-up
1	79	male	Nodular lymphocyte-predominant Hodgkin's lymphoma (perigastric lymph node)	Gastric adenocarcinoma, intestinal type	Gastrectomy	Death following gastrectomy
2	78	female	Classical Hodgkin's lymphoma-nodular sclerosis/mixed cellularity (cervical lymph node)	Salivary gland adenocarcinoma	Surgical resection	Lost from follow-up
3	48	female	Diffuse large B-cell non-Hodgkin's lymphoma (stomach and perigastric lymph node)	Gastric adenocarcinoma (diffuse type)	Gastrectomy + chemotherapy	30 months, alive
4	68	female	Splenic marginal zone lymphoma	Invasive ductal breast carcinoma	Mastectomy + splenectomy + chemotherapy	8 months, alive
5	71	male	Multiple myeloma	Papillary thyroid carcinoma	Clinical support	Death a few weeks after diagnosis of multiple myeloma
6	16	female	Classical Hodgkin's lymphoma-nodular sclerosis (cervical lymph node)	Papillary thyroid carcinoma	Thyroidectomy	22 months, alive
7	68	male	Extranodal gastric anaplastic large cell lymphoma	Pulmonary adenocarcinoma	Gastrectomy + chemotherapy	9 months, alive

## DISCUSSION

In the present report, seven cases of adenocarcinoma associated with lymphoma in lymph nodes are described, with analysis from surgical specimens. Second primary malignancies in patients with a history of malignant tumors have been reported with increasing frequency over recent years.^2^ Some combinations of tumors appear to have no readily detectable apparent common link or pathogenesis. Other combinations, in which lymphomas represent one of the components, have led to speculation concerning common underlying pathogenetic mechanisms that might contribute towards the development and growth of tumors.^1^

Various factors that might contribute towards the occurrence of multiple neoplasms have been suggested. Some authors have considered that the most important ones are genetic susceptibility (for example, mutations in the *P53* gene and defects in other genes responsible for DNA repair), advanced age and depressed cell immunity (immunosuppression produced by the first tumor or individual susceptibility).^2^ Other factors suggested in the literature have been growth factor production from the original tumor and exposure to common inducing agents, such as smoking or predisposition to carcinomas of the head and neck, lung, liver and bladder. Production of interleukin 6 in cases of renal carcinoma, for instance, has been reported to be a factor in the pathogenesis of multiple myeloma.^3^

Another association of tumors for which common pathogenesis is ascribed consists of gastric mucosa-associated lymphoid tissue (MALT-lymphoma) and gastric adenocarcinoma. *Helicobacter pylori* seems to be implicated in both of these entities.^4^ The frequency of *H. pylori* has been reported to be 45%-90% in patients with gastric adenocarcinoma, and 56%-100% in those with gastric MALT-lymphoma.^4^ On the other hand, *H. pylori* was found in 25%-38% of large B-cell gastric lymphomas.^4^ Case 3 of our series is peculiar, both because this bacteria was detected and because associations of diffuse-type gastric carcinoma with large B-cell lymphoma is reported to be extremely rare.^4^

Gastric adenocarcinoma concomitant with lymphocyte-predominant Hodgkin's lymphoma (our case 1) has not previously been described. An association between solid cancers and splenic marginal zone lymphoma (case 4) has already been reported.

The occurrence of collision tumors in lymph nodes infiltrated by lymphoma is exceptional. It is possibly due to obliteration of the lymphatic channels by the proliferating lymphoid cells, or to modification of adhesion molecules, thereby preventing circulation or implantation of metastatic cells. It has been also reported that neoplastic lymphoid cells could locally reduce tissue necrosis factor or interleukin-1-induced adhesion of neoplastic cells to the endothelial layer of lymph nodes.^3^

Multiple malignant tumors may occur in the context of hereditary cancer syndromes. They are derived from germline mutations of three major genes that are closely related to cell cycles, tumor suppression and differentiation, thereby potentially leading to carcinogenesis. These are oncogenes, such as the RET (rearranged during transfection) gene, which is responsible for multiple endocrine neoplasia type 2, and tumor suppressor genes, such as *P53*, which accounts for the Li-Fraumeni syndrome. Stability genes may also be affected, such as those involved in Fanconi's anemia.^5^ Patients with these syndromes frequently present concomitant neoplasms at specific sites, mostly at a younger age, with features differing from those of the seven cases reported in this study.

Although infrequent, the cases presented here should alert clinicians and pathologists to the possibility that concomitant neoplasms may exist, in order to prevent inaccurate or delayed diagnosis and staging. Lymph nodes should be carefully examined when searching for metastasis, because of the possibility of a second hematological malignancy. Lymph node biopsy in patients with a known primary malignancy does not always indicate metastasis.^2^ Diagnosis of such associations is critical because the therapies and prognoses are usually different. This type of association poses histopathological diagnostic difficulty.

## CONCLUSION

Whenever uncommon cell types or suspicious morphological features are seen in histopathological analysis of neoplasms or in suspect lymph nodes, the possibility of concomitant neoplasia should be considered. In difficult cases, immunohistochemical analysis is essential.
